# Enhanced primary ciliogenesis via mitochondrial oxidative stress activates AKT to prevent neurotoxicity in HSPA9/mortalin-depleted SH-SY5Y cells

**DOI:** 10.1186/s13041-023-01029-7

**Published:** 2023-05-11

**Authors:** Ji-Eun Bae, Soyoung Jang, Joon Bum Kim, Hyejin Hyung, Na Yeon Park, Yong Hwan Kim, So Hyun Kim, Seong Hyun Kim, Jin Min Ha, Gyeong Seok Oh, Kyuhee Park, Kwiwan Jeong, Jae Seon Jang, Doo Sin Jo, Pansoo Kim, Hyun-Shik Lee, Zae Young Ryoo, Dong-Hyung Cho

**Affiliations:** 1 Brain Science and Engineering Institute, Kyungpook National University, Daegu, 41566 Republic of Korea; 2 School of Life Sciences, BK21 FOUR KNU Creative BioResearch Group, Kyungpook National University, Daegu, 41566 Republic of Korea; 3 Bio-center, Gyeonggido Business & Science Accelerator, Suwon, Gyeonggido 16229 Republic of Korea; 4 Department of Bio-Medical Analysis, Bio Campus of Korea Polytechnic, Nonsan, Chungcheongnamdo 32943 Republic of Korea; 5 ORGASIS Corp., Suwon, Gyeonggido 16229 Republic of Korea

**Keywords:** HSPA9/mortalin, Primary cilia, Mitochondrial stress, Neurotoxicity, SH-SY5Y cells

## Abstract

The primary cilium, an antenna-like structure on the cell surface, acts as a mechanical and chemical sensory organelle. Primary cilia play critical roles in sensing the extracellular environment to coordinate various developmental and homeostatic signaling pathways. Here, we showed that the depletion of heat shock protein family A member 9 (HSPA9)/mortalin stimulates primary ciliogenesis in SH-SY5Y cells. The downregulation of *HSPA9* enhances mitochondrial stress by increasing mitochondrial fragmentation and mitochondrial reactive oxygen species (mtROS) generation. Notably, the inhibition of either mtROS production or mitochondrial fission significantly suppressed the increase in primary ciliogenesis in HSPA9-depleted cells. In addition, enhanced primary ciliogenesis contributed to cell survival by activating AKT in SH-SY5Y cells. The abrogation of ciliogenesis through the depletion of IFT88 potentiated neurotoxicity in *HSPA9*-knockdown cells. Furthermore, both caspase-3 activation and cell death were increased by MK-2206, an AKT inhibitor, in HSPA9-depleted cells. Taken together, our results suggest that enhanced primary ciliogenesis plays an important role in preventing neurotoxicity caused by the loss of HSPA9 in SH-SY5Y cells.

## Introduction

Cilia, which can be categorized into motile and non-motile primary cilia, are dynamically regulated but highly conserved organelles. In most cases, motile cilia have a 9 + 2 microtubule structural arrangement, whereas non-motile primary cilia have a 9 + 0 microtubule structural arrangement [1, 2]. Cilia are cellular organelles primarily composed of a membrane, soluble compartment, axoneme, basal body, and ciliary tip [1, 2]. Primary cilia are mechanical and chemical sensory organelles that play critical roles in sensing the extracellular environment to coordinate developmental and homeostatic signaling pathways [1, 2]. Primary cilia are implicated in the regulation of essential signal transduction mechanisms that control a wide variety of cellular events, including sonic hedgehog (Shh) [3], Wnt [4, 5], TGF-β [6], and platelet-derived growth factor (PDGF)-mediated cell signaling [7]. PDGF receptor alpha (PDGFRα) signaling in the primary cilium regulates the AKT (also known as protein kinase B/PKB) signaling pathway [8]. Furthermore, the dysregulation of primary cilia functions caused by the loss of ciliary proteins increases cell death, whereas enhanced primary ciliogenesis promotes cell survival under various stress conditions [9, 10]. Thus, disrupting the regulatory functions of cilia seems to underlie a diverse spectrum of human disorders, the primary ciliopathies. Ciliopathies include numerous seemingly unrelated developmental syndromes and manifest in the retina, kidney, liver, pancreas, skeletal system, and the brain [2, 11].

Structurally, the primary cilium is composed of functional domains, including the basal bodies, transition fibers, transition zone, intraflagellar transport (IFT) machinery, axoneme, and the ciliary membrane. IFT particles are large complexes comprising subcomplexes A and B, which mediate the bidirectional movement of protein cargo along the axonemal microtubules. Mutations in the IFT proteins of the cilia result in a group of inherited human diseases referred to as ciliopathies. IFT88, a core anterograde protein, is critical for cilia assembly and maintenance [12]. In addition to IFT proteins, ADP-ribosylation factor-like protein 13B (ARL13B) and Smoothened (Smo) are also mainly localized to the primary cilia. Thus, these proteins are commonly used as monitoring markers of primary cilia [3].

Recent studies have revealed a close relationship between primary cilia and mitochondrial dysfunction. The mTOR pathway associates cellular energy with mitochondrial biogenesis and ciliary length [13]. It has been found that mitochondrial dysfunction compromises ciliary homeostasis in astrocytes. Moreover, the loss of primary cilia promotes mitochondria-dependent apoptosis in thyroid cancer [10]. Our group recently showed that primary cilia mediate mitochondrial stress responses and autophagy to promote dopamine neuron survival in a Parkinson’s disease model [9]. Reactive oxygen species (ROS) also regulate primary cilium length during kidney injury caused by ischemia/reperfusion insult [14]. Hence, growing evidence indicates an interplay between primary cilia and mitochondrial function. Nonetheless, the mechanism underlying the interplay between primary cilia and mitochondria is still largely unknown.

In this study, we screened a mitochondrial protein library and found that the loss of heat shock protein family A (Hsp70) member 9 (HSPA9), also known as mortalin, induces primary ciliogenesis. Depletion of HSPA9 induces mitochondrial stress by increasing mitochondrial fragmentation and mitochondrial ROS (mtROS) generation. In addition, we found that enhanced primary ciliogenesis due to mitochondrial stress in HSPA9-deficient SH-SY5Y cells prevents neuronal cell death by activating AKT.

## Materials and methods

### Reagents and gene knockdown

Doxycycline (D9891) and N-acetylcysteine (NAC, A9165) were purchased from Sigma-Aldrich (St. Louis, MO, USA). 3-(2,4-dichloro-5-methoxyphenyl)-2,3-dihydro-2-thioxo-4(1 H)-quinazolinone (Mdivi-1, BML-CM127) was purchased from Enzo Life Sciences (Farmingdale, NY, USA). MK-2206 (S1078) was purchased from Selleck Chemicals (Houston, TX, USA). For gene expression knockdown, cells were transfected with validated small inhibitory RNAs (siRNAs) targeting human *HSPA9* (5′-AAACGCAAGUGGAAAUUAA-3′), *Drp1* (5′-GAGGUUAUUGAACGACUCA-3′), or *IFT88* (5′-CCGAAGCACUUAACACUUA-3′) using Lipofectamine 2000. The siRNAs were synthesized by Genolution (Seoul, Korea). At 48 h post-transfection, the cells were treated with the indicated reagents.

### Cell lines and primary cell culture

SH-SY5Y neuroblastoma cells were obtained from ATCC (Manassas, VA, USA). Human telomerase-immortalized retinal pigmented epithelial (RPE) cells were kindly provided by Dr. Jun Kim (KAIST, South Korea). TT cells stably expressing lentiviral doxycycline-inducible small hairpin RNA(shRNA) targeting *HSPA9* (TT/sh*HSPA9*) were kindly provided by J.I. Park (University of Wiscosin-Milwaukee, USA). To generate stable cell lines, SH-SY5Y cells were transfected with pMito-HyPer (SY5Y/Mito-HyPer) using Lipofectamine 2000, according to the manufacturer’s protocol (#11668019, Thermo Fisher Scientific, Waltham, MA). Positive transfectants were selected via growth in a medium containing 1 mg/mL G418 (#10131027, Thermo Fisher Scientific) for 7 days. After single-cell dropping, stable clones were selected under a fluorescence microscope (IX71, Olympus, Tokyo, Japan).

### Cilia staining and counting

For the staining of primary cilia, the cells were washed with cold phosphate-buffered saline (PBS) and fixed with 4% (w/v) paraformaldehyde, which was dissolved in PBS containing 0.1% (v/v) Triton X-100. Subsequently, cells were blocked with PBS containing 1% bovine serum albumin (BSA) and incubated overnight at 4 °C with primary antibodies against acetylated α-tubulin (1:1000; T7451, Sigma-Aldrich), γ-tubulin (1:1000; T5326, Sigma-Aldrich), and ARL13B (1:1000; 17711-1-AP, Proteintech) in 1% BSA. After washing, the cells were incubated with Alexa Fluor 488- or 555-conjugated secondary antibodies at room temperature (RT) for 1 h. Before mounting, the cells were treated with Hoechst 33,342 dye (1:10000, H3570, Thermo Fisher Scientific) for nuclear staining. Cilia were observed using a fluorescence microscope. Cilia were counted in approximately 200 cells for each experimental condition (n = 3). The ciliated cell percentage was calculated as follows: (total number of cilia/total number of nuclei in each image) × 100. Cilia lengths were measured using the free-hand line selection tool of Cell Sense Standards software (Olympus Europa Holding GmbH, Hamburg, Germany), and the average cilium length was calculated.

### Western blot analysis

Cell lysates were prepared in 2× Laemmli sample buffer [62.5 mM Tris-HCl, pH 6.8, 25% (v/v) glycerol, 2% (w/v) SDS, 5% (v/v) β-mercaptoethanol, and 0.01% (w/v) bromophenol blue] (#161–0737, Bio-Rad, Hercules, CA, USA). After separation via 10–12% SDS-PAGE, the proteins were transferred onto a PVDF membrane (#162–0177, Bio-Rad). The membranes were then incubated with the following primary antibodies: Drp1 (#611738, BD), IFT88 (13967-1-AP, Proteintech, Chicago, IL, USA), HSPA9 (Sc-13967, Santa Cruz Technology, CA, USA), Gli2 (18989-1-AP, Proteintech) p-AKT (#9271S, Cell Signaling Technology), cleaved caspase-3 (#9661S, Cell Signaling Technology), and actin (MAB1501, Millipore, Temecula, CA, USA). For protein detection, the membranes were incubated with the corresponding horseradish peroxidase (HRP)-conjugated secondary antibodies (Pierce, Rockford, IL, USA). Chemiluminescent signals were developed using Clarity Western ECL substrate (W3680-010, Bio-Rad).

### Determination of cellular ROS and mitochondrial ROS levels

Intracellular ROS levels were assayed using the fluorescent dye 2,7-dichlorofluorescein diacetate (DCFH-DA) (Invitrogen, Carlsbad, CA, USA), according to the manufacturer’s instructions. DCFH-DA is converted to the highly fluorescent compound 2,7-dichlorofluorescein (DCF) in the presence of oxidants. Briefly, SH-SY5Y cells were plated in 96-well plates, transfected with siRNA, and incubated with DCFH-DA (20 µM) in the presence or absence of N-acetyl-L-cysteine (NAC). The relative ROS ratios were measured using a fluorescence microplate reader (Victor X3, PerkinElmer, Waltham, MA, USA). The levels of mitochondria-specific ROS were assessed using the HyPer protein system. The pHyPer-dMito vector encoding mitochondria-targeted HyPer (Mito-HyPer) was obtained from Eyrogen (San Diego, CA, USA). SH-SY5Y cells stably expressing Mito-HyPer were transfected with scrambled or *HSPA9*-specific siRNAs for 72 h in the presence or absence of NAC or Mito-Q. The fluorescence intensities were monitored using a fluorescence plate reader (excitation 500 nm/emission 516 nm) (Victor X3) or fluorescence microscopy.

### Measurement of mitochondrial length

For the staining of mitochondria, cells were fixed with 4% PFA and then treated with a MitoTracker probe (100 nM, M7512, Thermo Fisher Scientific) for 30 min. Mitochondrial images were obtained using a fluorescence microscope (IX71; Olympus, Japan). The mitochondrial length was measured using the free-hand line selection tool of Cell Sense Standards software (Olympus Europa Holding GmbH). The mean length of the mitochondria was determined by selecting 20–30 linearized and unconnected filament-like mitochondria per cell using a tool provided by the Cell Sense Standards software (*n* = 3 independent experiments). Images of individual cells were analyzed and digitized using GraphPad Prism 8 (GraphPad Software, San Diego, CA, USA).

### Cell viability analysis

For the cell proliferation assay, SH-SY5Y cells seeded in 96-well plates were transfected with *HSPA9* siRNA. After transfection, the cell proliferation rate was measured daily for using a Cell Counting Kit-8 (CCK8) solution reagent (10 µM) (Dojindo Laboratories, Kumamoto, Japan) for 2 h, following the manufacturer’s instructions. Absorbance was measured using a spectrophotometer (Victor-X3, PerkinElmer).

### Statistical analysis

Statistical analyses of the results were performed using one-way analysis of variance (ANOVA) followed by a *post-hoc* least significant difference (LSD) test or an unpaired Student’s t-test using Origin software (San Clemente, CA, USA) or GraphPad Prism 8 (GraphPad Software, San Diego, CA, USA). Data were obtained from at least three independent experiments and are presented as the mean ± standard error of the mean (SEM). Statistical significance was defined as *p* < 0.05.

## Results

### Depletion of HSPA9 induces primary ciliogenesis

Recently, it has been shown that primary cilia are involved in mitochondrial stress and apoptosis [9, 10]. However, the interplay between the primary cilia and mitochondria remains unknown. To identify novel modulators of ciliogenesis in mitochondria, we screened a small siRNA library consisting of mitochondrial proteins using htRPE cells that stably expressed GFP-fused Smo (htRPE/Smo-GFP), which is widely used as a cilium marker. Based on this screening, we selected siRNAs for *HSPA9/mortalin* as a potent novel inducer of ciliogenesis. HSPA9 is primarily localized to the mitochondria and is involved in multiple mitochondrial processes, including energy metabolism, free-radical generation, and maintenance of mitochondrial protein integrity [15]. However, the role of HSPA9 in cilia has not been elucidated.

**Fig. 1:**
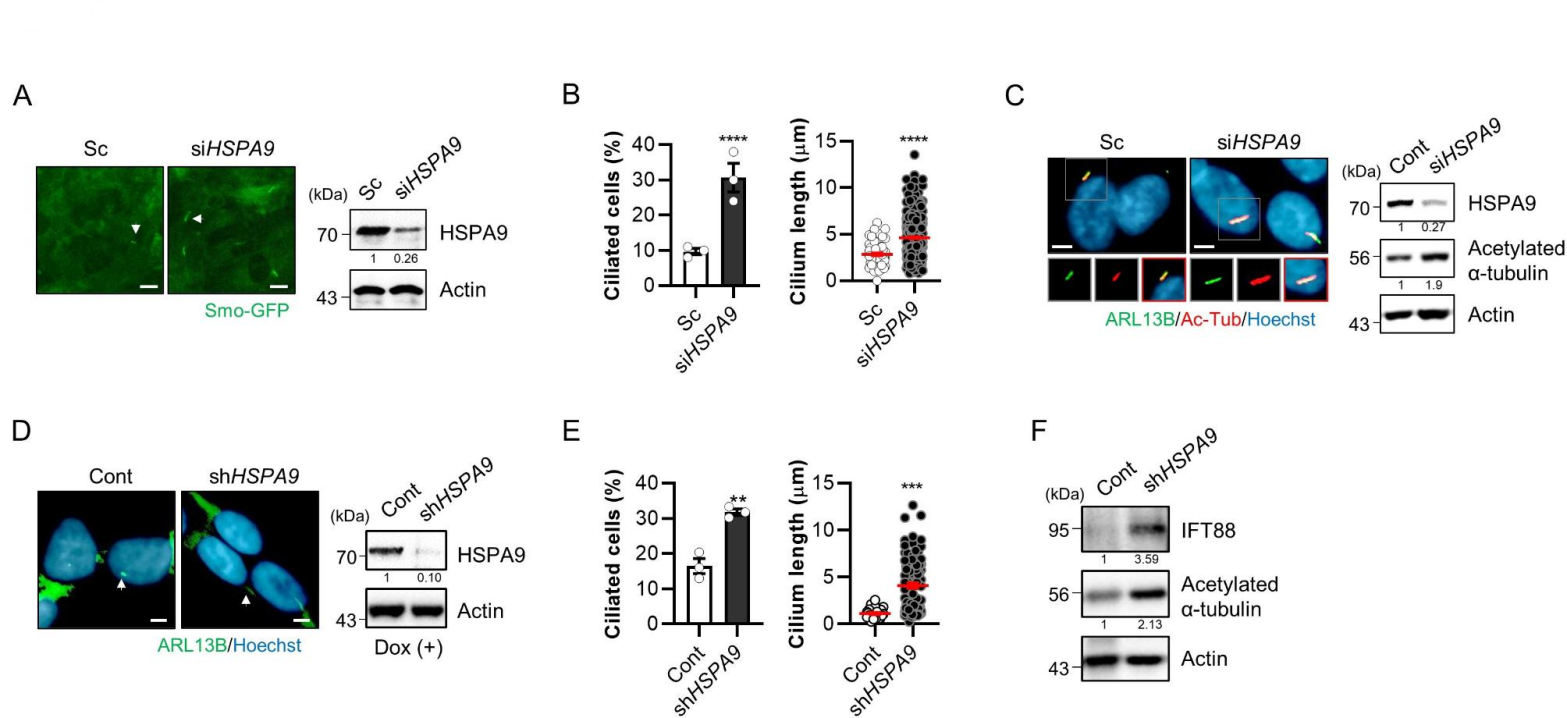
Depletion of HSPA9 promotes primary ciliogenesis. **(A, B)** htRPE/Smo-GFP cells were transfected with scrambled siRNA (Sc), or *HSPA9* siRNA (si*HSPA9*). After 3 days, the cells were imaged for Smo-GFP via fluorescence microscopy. Decreased expression of HSPA9 by RNA interference was confirmed via western blotting **(A)**. Scale bar: 10 μm. The ciliated cells and cilium length of the cells were measured **(B)**. (**C**) RPE cells were transiently transfected with Sc or si*HSPA9*, and primary cilia were stained with antibodies against ARL13B (green), acetylated α-tubulin (Ac-Tub, red). The nucleus (blue) was counterstained with Hoechst 33,342 dye. And the cells were analyzed by western blotting with indicated antibodies. Scale bar: 5 μm. **(D-F)** TT cells stably expressing pTRIPZ/sh*HSPA9* (TT/sh*HSPA9*) were treated with doxycycline (Dox) for 8 days. The cells were stained with ARL13B (green) and Hoechst 33,342 (blue) to count ciliated cells and cilium length **(D, E)**. Scale bar: 5 μm. TT cells treated with Dox were further analyzed by western blotting with anti-IFT88 and anti-acetylated α-tubulin antibodies **(F)**. Data were obtained from at least three independent experiments and the results are presented as the means ± S.E.M. (n = 3, ** *p* < 0.01, *** *p* < 0.005, **** *p* < 0.001)

**Fig. 2:**
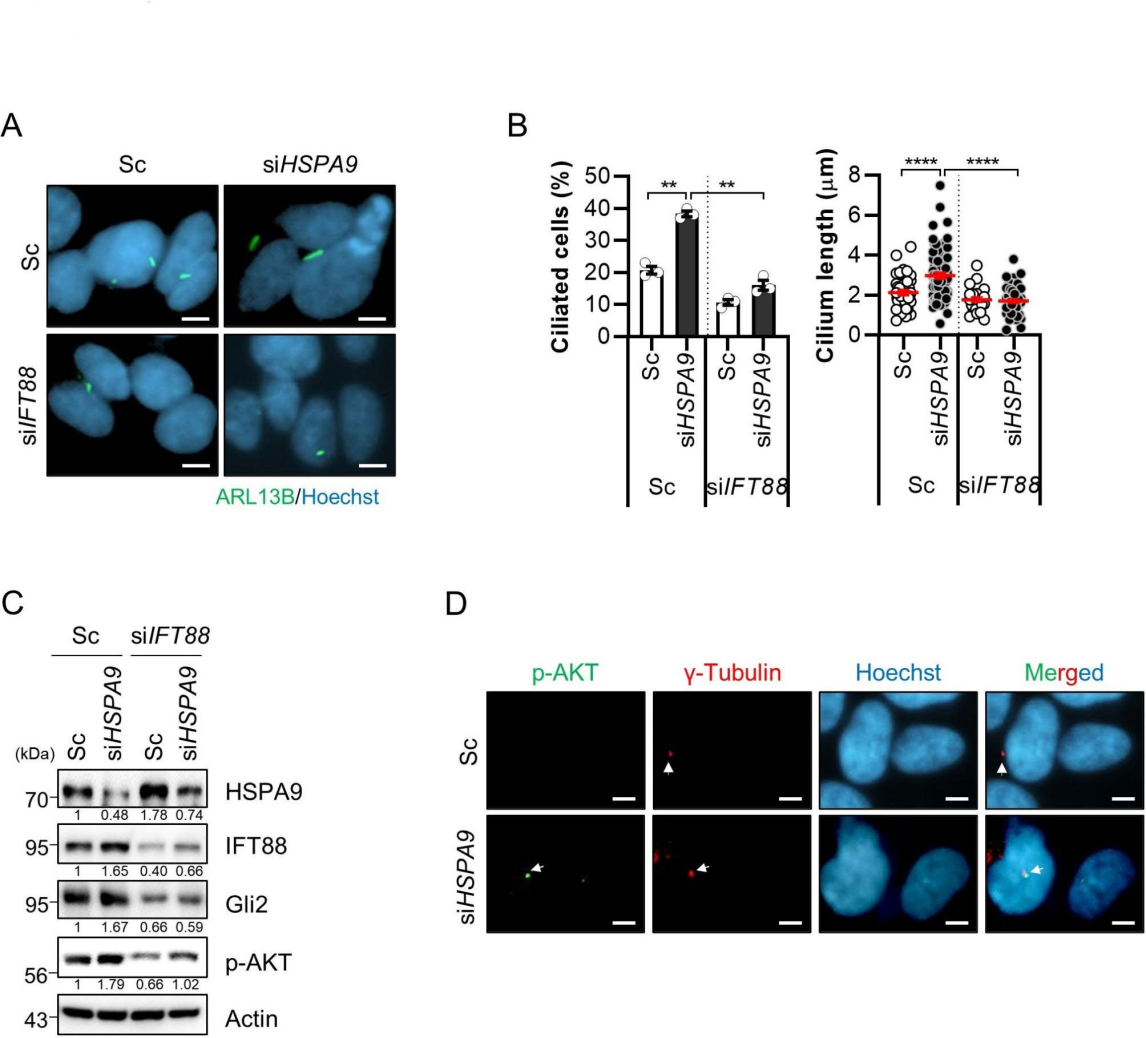
Loss of *IFT88* blocks primary ciliogenesis and ciliary signaling by depleting HSPA9 in SH-SY5Y cells. **(A-C)** SH-SY5Y cells transiently co-transfected with si*HSPA9* with or without *IFT88* siRNA (si*IFT88*) were imaged by staining with ARL13B antibody (green) and Hoechst 33,342 dye (blue) **(A)**. Primary cilia were also observed in the cells under a fluorescent microscope **(B)**. SH-SY5Y cells co-transfected with si*HSPA9* with or without si*IFT88* were analyzed via western blotting with the indicated antibodies **(C)**. (**D**) SH-SY5Y cells transiently transfected with scrambled siRNA (Sc) or si*HSPA9*. After 3 days, the cells were stained with phospho-AKT antibody (green), γ-Tubulin antibody (red), and Hoechst 33,342 dye (blue). Data were obtained from at least three independent experiments and the results are presented as the means ± S.E.M. (n = 3, ** *p* < 0.01, **** *p* < 0.001), Scale bar: 5 μm

To confirm the screening results, htRPE/Smo-GFP cells were transiently transfected with scrambled or specific siRNA against *HSPA9*. Consistent with the screening results, HSPA9 depletion resulted in enhanced primary cilia in htRPE cells (Fig. 1A, B). Tubulin undergoes various post-translational modifications, including acetylation and glutamylation, and these modifications are highly enriched in microtubules of the axoneme in primary cilium [16]. Thus, we further examined the levels of acetylated α-tubulin by depletion of HSPA9 in RPE cells. As shown in Fig. 1C, not only ARL13B but also acetylated α-tubulin were increased in *HSPA9* knockdown cells (Fig. 1C). Then the promotion of primary ciliogenesis was further confirmed using a doxycycline-induced shRNA knockdown system [17]. Consistent with previous report, treatment with doxycycline efficiently reduced the expression of HSPA9 in the TT/sh*HSPA9* cells (Fig. 1D). Subsequently, the primary ciliogenesis was enhanced by treatment with doxycycline in the TT/sh*HSPA9* cells (Fig. 1E, F). Previously, we showed that decreased HSPA9 expression is associated with neurodegenerative diseases, such as Alzheimer’s disease (AD) and Parkinson’s disease (PD) [18, 19]. Thus, we investigated whether HSPA9 affected primary ciliogenesis in SH-SY5Y neuroblastoma cells. Consistent with the effects observed in RPE cells, the primary cilium length was robustly increased after *HSPA9* knockdown in SH-SY5Y cells (Fig. 2A, B).

IFT88/polaris is a component of the IFT-B protein complex that mediates antegrade IFT, where a lack or hypomorphic mutation in *IFT88* disrupts the cilia assembly [20]. Thus, we further addressed the effect of HSPA9 on primary ciliogenesis by depleting IFT88 expression. According to a previous study, knockdown of the ciliary core protein *IFP88* blocked primary ciliogenesis in HSPA9-deficient SH-SY5Y cells (Fig. 2A, B). Interestingly, we found that the depletion of HSPA9 induced AKT phosphorylation, which was also blocked by *IFT88* knockdown (Fig. 2C), Recently, it was reported that phosphorylated AKT localizes at the ciliary base centrosome to recruit ciliary protein [21]. We next examined the localization of phospho-AKT in *HSPA9*-knockdown cells and found that phospho-AKT is co-localized with γ-Tubulin (Fig. 2D), suggesting that AKT is activated by promoting primary ciliogenesis in HSPA9-deficient cells. Taken together, these results suggest that the loss of *HSPA9* influences induction of primary ciliogenesis.

### Increased mitochondrial ROS levels influence primary ciliogenesis via the depletion of HSPA9 in SH-SY5Y cells

**Fig. 3:**
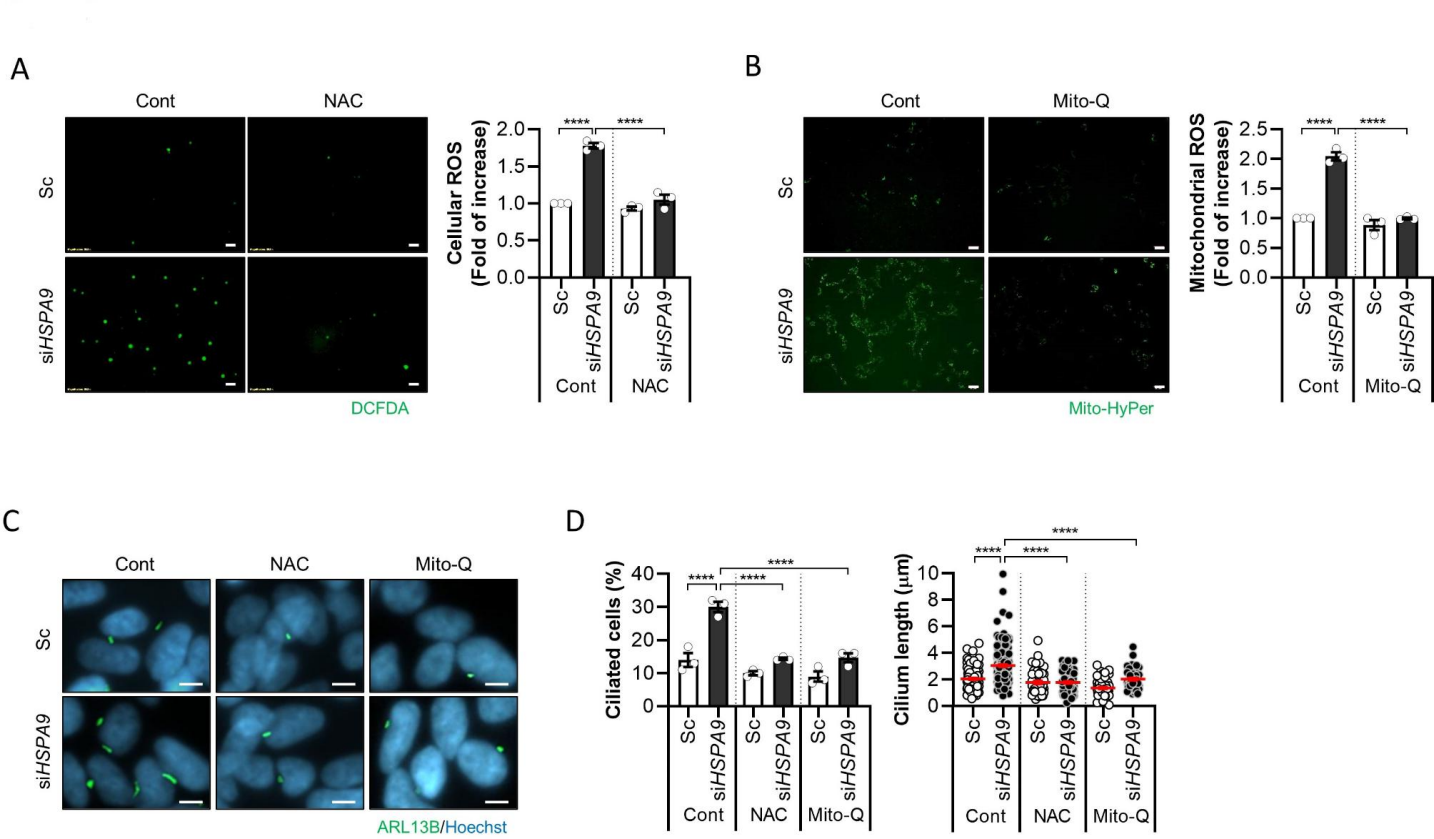
Increased mitochondrial ROS levels mediate primary ciliogenesis by depleting HSPA9 levels in SH-SY5Y cells. **(A)** SH-SY5Y cells transfected with scrambled siRNA (Sc) or siRNA against *HSPA9* (si*HSPA9*) for 3 days were further treated with N-acetylcysteine (NAC, 1 mM), and then incubated with DCFH-DA. The cells were imaged (*left*), and the fluorescence intensity of DCF was measured using a fluorescence microplate reader (*right*). **(B)** SY5Y/Mito-HyPer cells were transfected with Sc or si*HSPA9*, and then treated with Mito-Q (20 nM). The level of mitochondrial H_2_O_2_ was imaged (*left*; scale bar, 5 μm) and measured using the fluorescence intensity of Mito-HyPer (*right*). **(C, D)** SH-SY5Y cells were transfected with Sc or si*HSPA9* for 3 days and treated with or without NAC (1 mM) or Mito-Q (1 µM). The cells were then immunostained with ARL13B (green) and Hoechst 33,342 dye (blue). Representative cilia images are presented **(C)** (Scale bar, 5 μm). Cilia measurement data were obtained from approximately 200 cells per group **(D)**. The experiments were repeated at least three times. Data are presented as the mean ± SEM. (n = 3, **** *p* < 0.001)

Next, we elucidated the molecular mechanisms underlying HSPA9 depletion-induced primary ciliogenesis. Previously, we showed that HSPA9 deficiency increased the generation of ROS by increasing mitochondrial fragmentation, which promoted primary ciliogenesis [16]. Therefore, we investigated the effect of *HSPA9* knockdown on ROS generation in SH-SY5Y cells. Cellular ROS levels were considerably increased upon *HSPA9* knockdown in SH-SY5Y cells; however, excessive ROS was removed after treatment with N-acetylcysteine (NAC), an ROS scavenger (Fig. 3A). Since dysfunctional and fragmented mitochondria generate higher levels of ROS, we further analyzed mtROS using the expression of a mitochondrial hydrogen peroxide sensor (mt-HyPer). *HSPA9* knockdown potently increased mtROS levels in SH-SY5Y cells. In addition, treatment with the mitochondria-targeted antioxidant drug mitoquinone (Mito-Q) blocked mtROS overproduction caused by the loss of *HSPA9* in SH-SY5Y cells (Fig. 3B). Notably, we found that *HSPA9* knockdown-induced primary ciliogenesis was strikingly suppressed upon treatment with the ROS scavengers NAC and Mito-Q (Fig. 3C, D), suggesting that mtROS critically mediates mitochondrial ROS-induced ciliogenesis by depleting HSPA9 in SH-SY5Y cells.

**Fig. 4:**
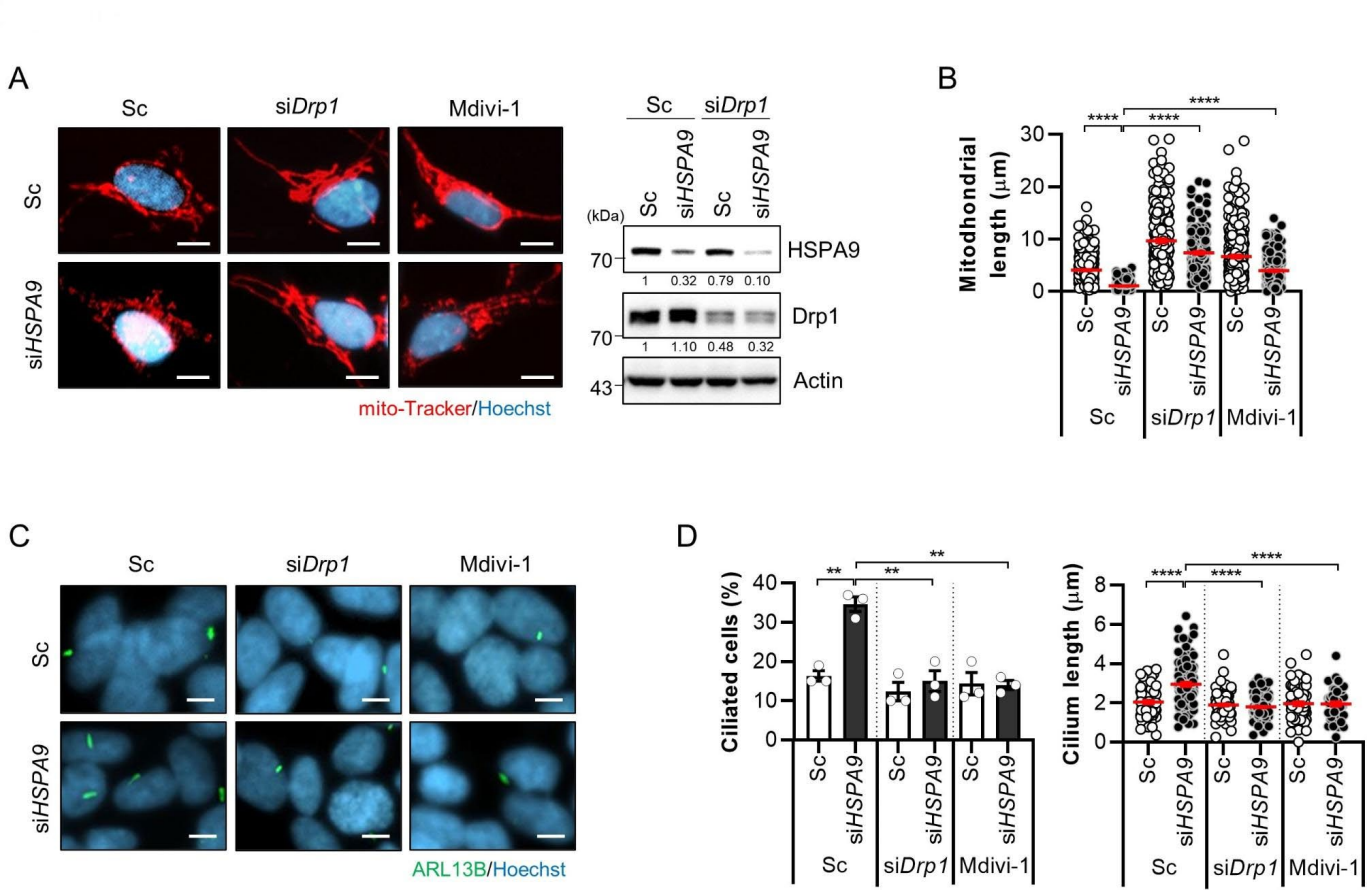
Inhibition of mitochondrial fission suppresses primary ciliogenesis in HSPA9-depleted SH-SY5Y cells. **(A, B)** SH-SY5Y cells were transfected with *HSPA9* (si*HSPA9*) with or without *Drp1* siRNA (si*Drp1*) for 3 days or treated with Mdivi-1 (10 µM) for 24 h. Mitochondrial fragmentation was confirmed using a Mito-tracker probe (red), and the cells were analyzed via western blotting with the indicated antibodies **(A)** (Scale bar, 20 μm). The mitochondrial length was further measured **(B)**. **(C, D)** SH-SY5Y cells transfected with *HSPA9* siRNA (si*HSPA9*) were transfected with *Drp1* siRNA (si*Drp1*) or treated with Mdivi-1 (10 µM). The cells were then immunostained with ARL13B (green) and Hoechst 33,342 dye (blue) **(C)** (Scale bar, 5 μm). Ciliated cells and the cilium length were measured in approximately 200 cells per group **(D)**. The experiments were repeated at least three times. Data are presented as the mean ± SEM. (n = 3, ** *p* < 0.01, **** *p* < 0.001)

Significant mitochondrial fragmentation is induced by excessive mtROS production, and we previously reported that the knockdown of *HSPA9* increases mitochondrial fission in a neurodegenerative disease model [18]. Thus, we investigated a possible link between mitochondrial fission/fusion and ciliogenesis following HSPA9 depletion. Mitochondrial dynamics are promoted by the GTPase family protein dynamin-1-like protein (Drp1) [22]. Consistent with previous reports, mitochondrial staining showed that *HSPA9* knockdown promoted mitochondrial fragmentation in SH-SY5Y cells (Fig. 4A). In addition, either genetic or chemical inhibition of *Drp1* by RNA interference or Mdivi-1 treatment, respectively, efficiently blocked mitochondrial fragmentation in HSPA9-depleted cells (Fig. 4A, B). We further explored the effect of inhibiting mitochondrial fragmentation on primary ciliogenesis. Notably, both genetic and chemical inhibition of Drp1 significantly suppressed ciliated cells and cilium length in HSPA9-depleted cells (Fig. 4C, D). Taken together, these results suggest that mitochondrial ROS and fission regulate primary ciliogenesis induced by *HSPA9* loss in SH-SY5Y cells.

### Enhanced primary ciliogenesis prevents neuronal cell death by activating AKT in HSPA9-depleted SH-SY5Y cells

**Fig. 5:**
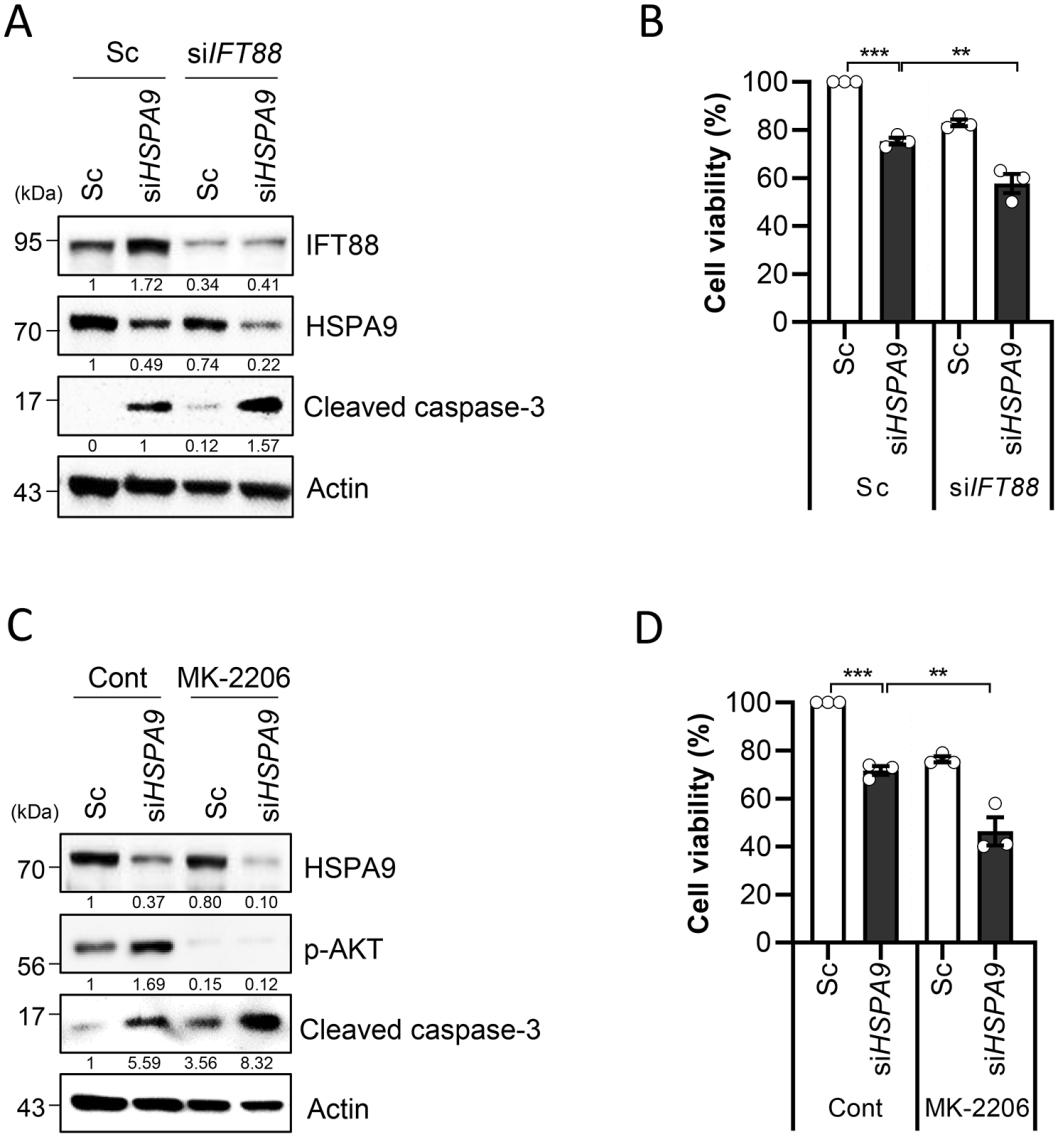
Primary cilia prevent neurotoxicity by activating AKT in HSPA9-depleted SH-SY5Y cells. **(A, B)** SH-SY5Y cells co-transfected with si*HSPA9* with or without si*IFT88* were analyzed via western blotting with the indicated antibodies **(A)**. Cell viability was determined through the CCK-8 assay. **(C, D)** SH-SY5Y cells transiently transfected with scrambled siRNA (Sc) or si*HSPA9* were treated with an AKT inhibitor, MK-2206 (5 µM), for 24 h. The cells were then harvested to analyze protein expression via western blotting **(C)** and to determine cell viability **(D)**. Data were obtained from at least three independent experiments and the results are presented as the means ± S.E.M. (n = 3, ** *p* < 0.01, *** *p* < 0.005)

Excessive mitochondrial fission has been implicated in a wide range of human disorders by increasing the susceptibility of cells to mitochondrial stress and apoptosis [23, 24]. In contrast, primary cilia can promote cell survival under various stress conditions, including oxidative stress [9]. In addition, the inhibition of HSPA9 has been shown to induce mitochondrial stress and cancer cell death [15, 25, 26]. Thus, we further addressed the effects of blocking enhanced primary ciliogenesis on HSPA9 deficiency-induced cell death. As shown in Fig. 5A, B, the downregulation of HSPA9 alone slightly induced the cleavage of caspase-3 and cell death in SH-SY5Y cells. However, the loss of primary cilia by co-knockdown with IFT88 further increased cell death and caspase-3 activation (Fig. 5A, B). Previously, we found that the downregulation of HSPA9 increased the phosphorylation of AKT/PKB, which promoted survival of SH-SY5Y cells. Thus, we further evaluated the role of AKT activation in cell death through the depletion of HSPA9. When IFT88 was depleted, both caspase-3 activation and cell death were enhanced after treatment with the selective AKT inhibitor MK-2206, compared to depletion of HSPA9 alone (Fig. 5C, D), indicating that enhanced primary ciliogenesis after *HSPA9* knockdown promotes cell survival by activating AKT in SH-SY5Y cells.

## Discussion

In this study, we found that the loss of *HSPA9/mortalin* induced primary ciliogenesis in SH-SY5Y cells. HSPA9 is a multipotent stress response chaperone protein induced by metabolic stress, glucose deprivation, calcium ionophores, thyroid hormone treatment, hyperthyroidism, ionizing radiation, and several cytotoxins [15]. Thus, it has been implicated in various cellular functions, including stress response, cell proliferation control, and apoptosis inhibition. In particular, HSPA9 regulates the functions of the tumor suppressor protein p53 and plays an important role in the stress response and maintenance of the mitochondria [17, 27]. However, the role of HSPA9 in primary ciliogenesis has not been elucidated.

We recently reported an interplay between two seemingly irrelevant organelles, primary cilia and mitochondria [9]. Mitochondrial stress-induced ciliogenesis is mediated by mtROS generation, which subsequently activates AMP-activated protein kinase (AMPK) and autophagy. However, the abrogation of ciliogenesis compromises mitochondrial stress-induced autophagy, leading to enhanced cell death in vitro and in vivo [9]. We further reported that the loss of *HSPA9* in AD models potentiates mitochondrial dysfunction by increasing mtROS generation and mitochondrial fission [18]. Based on these previous reports, we confirmed that HSPA9 depletion increased mtROS and mitochondrial fragmentation (Figs. 3 and 4). In addition, our data suggest that the depletion of HSPA9 induced primary ciliogenesis in SH-SY5Y cells (Figs. 1 and 2). Notably, we also found that disruption of cilium by *IFT88* knockdown upregulated the expression of HSPA9 (Figs. 2C and 5 A). Although the effect of impaired primary cilium on HSPA9 function has not been elucidated, recent reports suggest that loss of *IFT88* potentiates mitochondrial dysfunctions including decrease of oxidative phosphorylation reaction and increase of mitochondrial fragmentation in various cells [28, 29]. Therefore, the crosstalk between the expressional regulation of HSPA9 and IFT88 will be further elucidated. Moreover, our results indicated that HSPA9 depletion enhanced primary ciliogenesis to suppress neurotoxicity during mitochondrial stress, decreasing mtROS levels and mitochondrial fission (Figs. 3 and 4).

The enhanced ciliated cells and cilium length were completely abolished by inhibiting either mtROS or mitochondrial fission in SH-SY5Y cells. Our findings indicate that ciliogenesis was enhanced in response to mitochondrial stress in SH-SY5Y cells. Furthermore, the cytoprotective effect of primary cilia is mediated, at least in part, through the activation of AKT in SH-SY5Y cells (Fig. 5). Our findings suggest that ciliogenesis may be an important adaptive or defensive mechanism against mitochondrial stress insults in neuronal cells. AKT is one of the most well-characterized kinases because of its critical role in regulating numerous cellular functions, including metabolism, development, proliferation, transcription, protein synthesis, and cell survival [30]. Although the precise mechanism of the ciliary signals that maintain the connectivity and viability of neurons remains unknown, primary cilia and ciliary signals play various essential roles during the development of the brain and central nervous system. Notably, it has been reported that primary cilia prevent environmental stress-induced dendritic degeneration by activating AKT signaling, which inhibits caspase-3 activation in the neonatal mouse forebrain [31]. Bowie and Goetz showed that primary cilia with TTBK2 are essential for the survival and connectivity of cerebellar Purkinje neurons [32]. Accordingly, we also found that enhanced primary cilia prevented neurotoxicity by activating AKT in HSPA9-depleted cells (Fig. 5). The loss of primary cilia additionally induced caspase-3 activation and cytotoxicity in HSPA9-depleted SH-SY5Y cells (Fig. 5A, B). In addition, both caspase-3 and cell death were significantly potentiated by AKT inhibition (Fig. 5C, D).

Recently, our group also reported that the loss of *HSPA9* induces peroxisomal autophagy (pexophagy) by increasing peroxisomal stress in neuronal cells [19]. Excessive HSPA9 depletion generates ROS to promote pexophagy and mitochondrial autophagy (mitophagy) [19, 33]. The ectopic expression of several loss-of-function mutants of *HSPA9* found in patients with PD and HSPA9-deficient cells failed to rescue mitochondrial dysfunction and phagocytic activity [19, 33]. Mitochondrial and peroxisomal dysfunction play major roles in the pathogenesis of PD [19, 34]. PD-associated mitochondrial dysfunction can result from diverse causes such as impaired mitochondrial biogenesis, increased ROS and mitochondrial fission, and defective mitophagy [34]. As mitochondrial stress-induced ciliogenesis is mediated by mtROS, which activates autophagy, further exploration of the role of HSPA9 in the regulation of primary ciliogenesis will be helpful in understanding neurodegenerative diseases, including PD. Taken together, our present findings extend the knowledge of HSPA9 in the repertoire of cilia-related biological functions and diseases.

## Data Availability

All of the data generated and analyzed in this study are included in this published article.

## Abbreviations

HSPA9: heat shock protein family A member 9
mtROS: mitochondrial reactive oxygen species
Shh: sonic hedgehog
PDGF: platelet-derived growth factor
IFT: intraflagellar transport (IFT)
ARL13B: ADP-ribosylation factor-like protein 13B
Smo: smoothened
NAC: N-acetyl-L-cysteine
RPE: retinal pigmented epithelial
shRNA: small hairpin RNA
HRP: horseradish peroxidase
DCFH-DA: 2,7-dichlorofluorescein diacetate
HyPer: hydrogen peroxide
Mito-Q: mitoquinone
Drp1: GTPase family protein dynamin-1-like protein
AMPK: AMP-activated protein kinase
Pexophagy: peroxisomal autophagy
